# Understanding of Contradiction on Concentration Effect on Stability, Physical Properties, Evaporation and Microexplosion Characteristics of Al/JP-10/Oleic Acid Nanofluid Fuel

**DOI:** 10.3390/nano12193446

**Published:** 2022-10-01

**Authors:** Qianmei Yang, Shengji Li, Linhui Ye, Xuefeng Huang

**Affiliations:** 1 College of Materials and Environmental Engineering, Hangzhou Dianzi University, Hangzhou 310018, China; 2 Department of Physics, Institute of Energy, Hangzhou Dianzi University, Hangzhou 310018, China

**Keywords:** nanofluid fuels, jet fuel, Al nanoparticles, evaporation, microexplosion

## Abstract

An Al/JP-10/oleic acid nanofluid fuel system has demonstrated potential in advanced combustion for aviation turbine engines. To improve the energy density of nanofluid fuel, a higher Al concentration requirement needs to be met. Correspondingly, a higher surfactant oleic acid concentration is required to maintain better dispersion stability. The increment of Al and oleic acid concentrations results in more frequent microexplosions, but a slower evaporation rate. Therefore, this paper proposes to deeply understand the contradiction of the concentration effect on the stability, physical properties, evaporation and microexplosion characteristics and obtain the best Al and oleic acid concentrations to maintain the most suitable comprehensive performance. Experiments on the stability, physical properties, evaporation and microexplosion characteristics were conducted, respectively. The analysis and discussion were then made to reveal the Al and oleic acid concentration effect on the stability, physical properties, evaporation and microexplosion characteristics. The results show that the optimum mass ratio of Al:oleic acid is 1:2 for the nanofluid fuels with Al concentrations of 2.5 wt.% or below, 1:2.5 for 5.0 wt.% or above to obtain the best stability. The physical properties of the nanofluid fuels such as density, surface tension and viscosity are linear, quartic and quadratic functions of Al concentration, respectively, relating to the internal flow and microexplosion of fuel droplets. With increasing oleic acid and Al concentration, the evaporation rates reduced, and the microexplosions became more frequent and intense. At a high ambient temperature of 600 °C, the evaporation rates were kept almost equivalent for JP-10, JP-10/oleic acid, and Al/JP-10/oleic acid fuels. It was found that the increment of ambient temperature can compensate for the reduction of the evaporation rate owing to the addition of oleic acid and Al nanoparticles, improving the evaporation and microexplosion performance.

## 1. Introduction

Jet fuel, consisting of complex hydrocarbon mixtures, has been designed and widely applied in aircraft powered by aviation turbine engines [1]. Additionally, jet fuel behaves as a fuel at ordinary pressure owing to its low oxygen content and reactivity, which has been identified as a potential jet propellant [2]. In general, the utilization of jet fuel mainly experiences the consecutive steps including atomization, evaporation, mixing, ignition and combustion [3,4]. To further enhance the utilization efficiency of jet fuel, jet fuel-based nanofluid fuels have been proposed through improving the evaporation and combustion performance. Nanofluid fuels are defined as a colloidal suspension of metallic nanoparticles in the base fuels [5]. Owing to the excellent advantages, such as high energy density, high probability of ignition, high combustion efficiency, high heat conductivity and low pollutant emissions [6,7,8], nanofluid fuels may have practical engineering application prospects and could even change the global energy landscape [9,10].

Among the jet fuels, JP-10 fuel (C_10_H_16_, exotetrahydrodicyclopentadiene), as a single-component advanced jet fuel, has been considered as a superfuel for military jets, missiles and supersonic combustion ramjets [11]. JP-10 fuel possesses many attractive advantages such as high energy density and good thermal stability over conventional jet fuels, e.g., the volumetric heat of 39.6 MJ/L, freezing point of <−100 °C, and kinematic viscosity of 19 cSt at −40 °C. [12]. To further improve the utilization efficiency of JP-10, an available method is to prepare JP-10-based nanofluid fuel through adding nanoparticles. These added nanoparticles can be mainly classified to three groups [13,14,15]: (1) metallic nanoparticles (Al, B, Ce, etc.); (2) metal oxide nanoparticles (Al_2_O_3_, CeO_2_, Fe_3_O_4_, TiO_2_, ZnO, CuO, NiO, etc.); and (3) carbon-based nanoparticles (graphite, carbon nanotube, graphene and graphene oxide). Al nanoparticles are one of the energetic metal fuels and have been widely used in propellants as an additive [16,17]. Therefore, in this work, an Al/JP-10/oleic acid nanofluid fuel was selected to evaluate the effects of nanoparticles on the stability, physical properties, evaporation and microexplosion characteristics.

The effect of the addition of Al nanoparticles on the evaporation, ignition and combustion performances of the base fuels has been investigated. Javed et al. [18] demonstrated the combustion characteristics of heptane droplets with different mass fractions of Al nanoparticles at 600–850 °C, and found that at 600–700 °C, the addition of 0.5% Al nanoparticles shortened the ignition delay time of the droplets, while the addition of Al nanoparticles of 2.5% and 5.0% prolonged the ignition delay time. For Al/kerosene nanofluid fuel [19,20], it was found that the ignition delay and ignition temperature of droplets with low Al concentrations (0.1%, 0.5% and 1.0%) decreased at 400–800 °C. The microexplosion phenomenon occurred in the droplets containing high Al concentrations (2.5%, 5% and 7.0%) and the combustion rate increased at 700–800 °C compared with the counterpart (base fuel). However, at 400–500 °C, the evaporation rate is generally equivalent to pure kerosene. Yu et al. [21] demonstrated that the addition of Al nanoparticles into JP-10 increased the combustion efficiency by 3.0–9.0%. However, the promotion or deterioration of evaporation and burn rates by adding nanoparticles and surfactants are controversial, and no complete systems or models have been developed to correctly describe the aforementioned relationship and elucidate the concentration effect.

The Al/JP-10/oleic acid nanofluid fuel system has been sparsely investigated. The detailed concentration effect of adding Al nanoparticles on the stability, physical properties, evaporation and microexplosion performances of JP-10 has not been demonstrated and its promotion or deterioration mechanism has not been fully revealed. Therefore, the main aims of this paper are to: (1) examine the Al concentration effect on the stability and physical properties including density, surface tension, and dynamic viscosity of prepared Al/JP-10/oleic acid nanofluid fuels, and then compare them with the properties of JP-10; and (2) obtain the critical Al concentration affecting the evaporation and microexplosion behavior and characteristics at the optimum surfactant concentration at different ambient temperatures, and further reveal the influence and mechanism of the addition of Al nanoparticles on the evaporation performance of JP-10.

## 2. Materials and Experimental Methods

### 2.1. Materials

**Figure 1:**
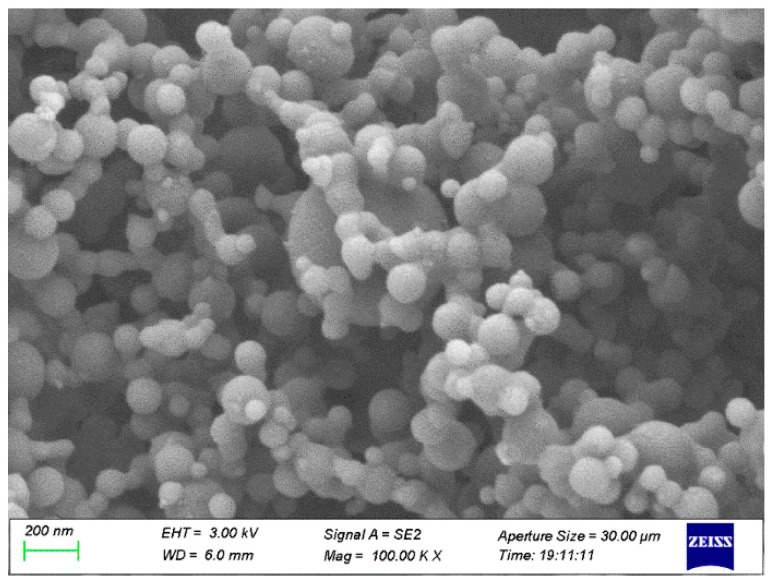
SEM morphology of Al nanoparticles.

**Figure 2:**
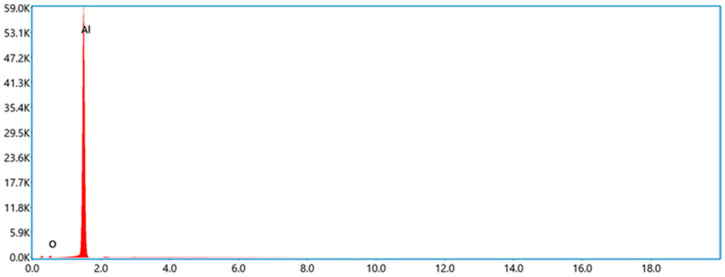
EDS spectrum of Al nanoparticles.

JP-10, one of the jet fuels, was obtained from Tianjing University, Tianjing, China. Its boiling point is ~156 °C. Al powder was commercially purchased from Dk Nano Technology Co., Ltd., Beijing, China, which was fabricated using the plasma vapor deposition technique. The morphology of the Al nanoparticles was characterized by scanning electron microscope (SEM, Sigma 300, Zeiss, Oberkochen, Germany), as shown in Figure 1. It looks spherical, and the median diameter is ~50 nm, approximately keeping agreement with the announced values. The Al and O atomic percentages of the Al nanoparticles (Figure 2), achieved by energy dispersive spectroscopy (EDS, Smart EDX, Zeiss, Oberkochen, Germany) analysis, are 98.60% and 1.40%, respectively. It is suggested that the passivation of Al nanoparticles after synthetization produces a thin layer of Al_2_O_3_ to prevent the surfaces of the Al nanoparticles from becoming oxidized. The volume density is 230 kg/m^3^ according to powder density tester analysis. The specific surface area of the Al powder is ~20 m^2^/g based on Brunauer, Emmett and Teller (BET) analysis. The surfactant oleic acid, purchased from Maclean Biochemical Technology Co., Ltd., Shanghai, China, was used to improve the stability of the Al nanoparticles suspended into JP-10. Its density and boiling point are 890 kg/m^3^ and ~195 °C, respectively.

### 2.2. Preparation of Al/JP-10/Oleic Acid Nanofluid Fuels

The preparation of nanofluids plays a significant role in their stability. Generally, the preparation of nanofluids usually adopts one-step and two-step methods [22]. The one-step method is to prepare nanoparticles simultaneously with their dispersion in the base liquid, and the two-step method is to separate the preparation of nanoparticles from the dispersion in the base liquid. The nanofluid prepared by the one-step method has better stability, but a higher cost. In this experiment, a two-step method was used to prepare the Al/JP-10/oleic acid nanofluid fuels. First, the Al powder and oleic acid were weighed, respectively. The Al powder was put into the oleic acid and then oscillated using an ultrasonic oscillator for 1 h, so that the oleic acid was fully wrapped onto the surface of the Al nanoparticles. Second, the mixtures with different mass ratios of Al and oleic acid were added into the JP-10 measured using a volumetric cylinder, and then oscillated by the ultrasonic oscillator for 20 min to form uniformly dispersive suspensions. It is worthy of note that the samples needed to be kept in an ice bath throughout the whole process of ultrasonic oscillation, and the temperature was controlled at 15 ± 5 °C, to achieve better stability.

### 2.3. Stability and Physical Property Test Method

The stability was tested using the dynamic light scattering (DLS, Nanotrac Flex, Microtrac, Montgomeryville and York, PA, USA) method, also known as photon correlation spectroscopy. It is a technique that uses the photon correlation principle to detect the small translation of scattered light due to Brownian motion and the Doppler effect to obtain the dynamic behavior information of the scattered particles. The photon correlation spectrum obtains the light intensity autocorrelation function from the scattering intensity fluctuation. The average attenuation constant of the correlation function is directly related to the average diffusion coefficient (*D_mean_*), that is, *D_mean_* can be obtained from the average attenuation constant of the correlation function. According to the Stokes–Einstein formula, the average hydration diameter *d_mean_* of the particles is inversely proportional to *D_mean_*, i.e.,

(1) $$d_{mean}=\frac{k_{B}T}{3\pi \mu D_{mean}}$$

where *k_B_* is the Boltzmann constant, *T* is the temperature, and *μ* is the viscosity.

The physical properties of Al/JP-10/oleic acid nanofluid fuels, such as density, surface tension and dynamic viscosity, were measured by the standard density bottle, Du Noüy Ring Tensiometer and Ostwald viscometer, respectively [23].

### 2.4. Experimental Setup for Evaporation

**Figure 3:**
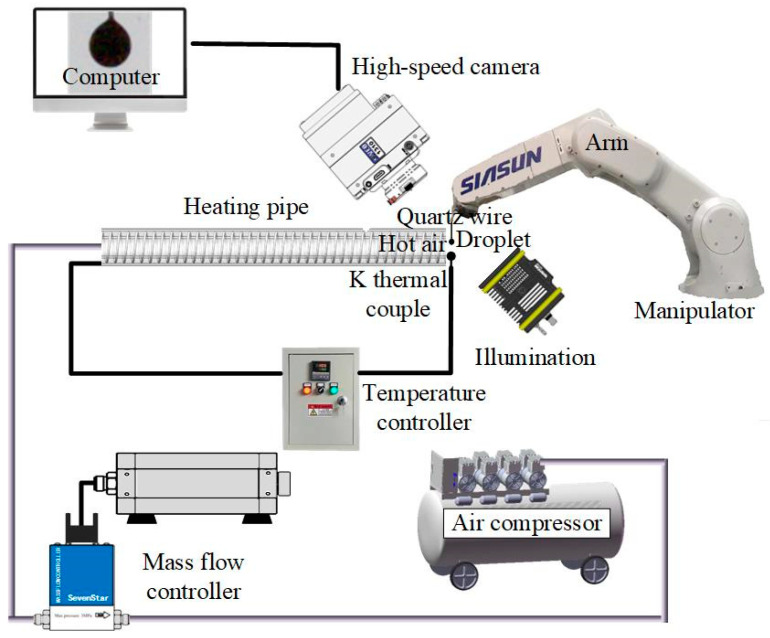
Schematic of experimental setup.

A self-built experimental setup demonstrated in our previous work [15] was used to conduct the test of evaporation characteristics, including a hot air supply module, a droplet suspension and position module, a high-speed imaging module, and an illumination module. A schematic of the experimental setup is shown in Figure 3. The hot air supply module provided a laminar air flow with a set temperature to heat the droplets. The droplet suspension and position module guarantees the droplets stay in the same heating zone. The droplets were suspended on quartz wire with a diameter of 125 μm. The droplet diameter was kept ~1.0 mm. The high-speed camera (Phantom M310, Vision Research Inc., Wayne, NJ, USA) with a lens (AT-X M100 AF PRO, Tokina, Japan) was used to record the videos at a frame ratio of 10,000 fps during the evaporation of the droplets. The illumination module provides a backlight illumination from an LED lamp and a condenser for clear high-speed imaging. The videos and pictures were treated using the digital imaging treatment method to measure the diameters of the droplets [3].

## 3. Results and Discussion

### 3.1. Effect of the Mass Ratio of Al and Oleic Acid on the Stability of Al/JP-10/Oleic Acid Nanofluid Fuels

**Figure 4:**
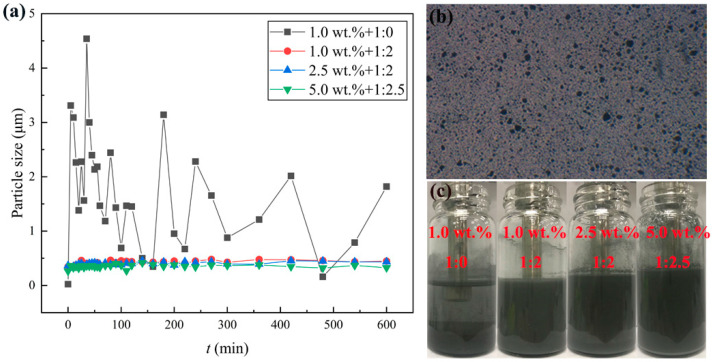
Stability of nanofluid fuels. (**a**) Particle size varies in the suspensions, (**b**) microscopic picture, (**c**) settling pictures after 9 h.

The mass ratios of Al to oleic acid are set to 1:0, 1:0.5, 1:1, 1:1.5, 1:2, 1:2.5 and 1:3, respectively. The mass ratio of 1:0 represents that the surfactant oleic acid was not added into the suspensions. Figure 4a shows that the size of particles in the suspensions without the oleic acid significantly fluctuated for 10 h, much larger than the median diameter of the Al nanoparticles, and the largest particle size reached 4.5 μm. It means that Al nanoparticles without the oleic acid presented severe agglomeration and sedimentation. The particle sizes of other suspensions with different oleic acid concentrations are 2~3 times greater than single Al nanoparticles. This may be attributed to the agglomeration among the Al nanoparticles owing to the interaction of the van der Waals’ force and Coulomb force among the nanoparticles. It can be testified that the fluctuation amplitude of particle size with the surfactant is obviously lower than that without the surfactant. Therefore, the addition of the surfactant oleic acid can improve the dispersion stability of the suspensions, lasting for over 10 h.

The aggregation of the Al nanoparticles can be observed by an optical microscope. Figure 4b demonstrates a microscopic picture of Al nanoparticles dispersed into JP-10 with 1.0 wt.% Al concentration and the mass ratio of Al to oleic acid at 1:2. It can be found that the Al nanoparticles were evenly dispersed in the suspension at the initial stage, but gradually, some of the Al nanoparticles aggregated and became enlarged. As the Al concentrations in the suspensions are different, the requirement of oleic acid is different. To obtain the best stability, for the suspensions of 1.0 wt.% and 2.5 wt.% Al concentrations, the mass ratio of Al to oleic acid should be 1:2; for 5.0 wt.% and even 10.0 wt.%, and the mass ratio should be adjusted to 1:2.5. Figure 4c shows a group of comparative pictures of suspensions after settling for 9 h, of 1.0 wt.% + 1:0, 1.0 wt.% + 1:2, 2.5 wt.% + 1:2, 5.0 wt.% + 1:2.5, from left to right, respectively. It suggests that the nanofluid fuels can obtain the best stability at the suitable mass ratios of Al and oleic acid.

### 3.2. Effect of Al Concentration on Physical Properties of Al/JP-10/Oleic Acid Nanofluid Fuels

**Figure 5:**
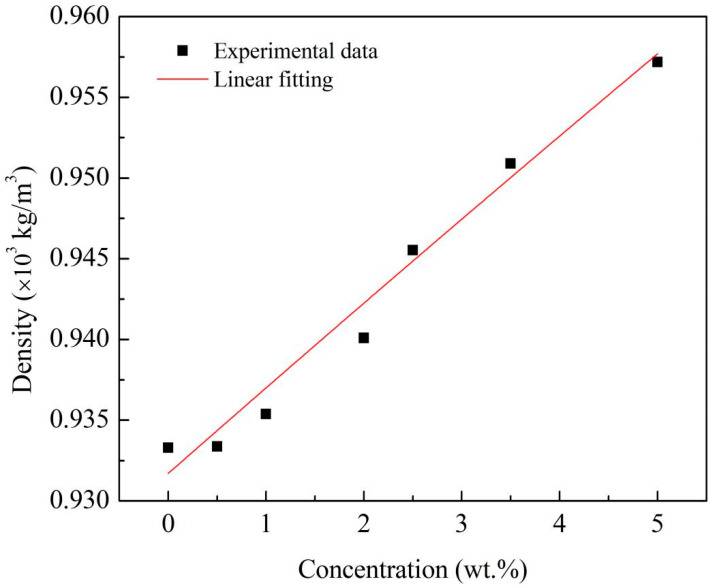
Density of Al/JP-10/oleic acid nanofluid fuels at different Al concentrations.

The measurements of the density of the Al/JP-10/oleic acid nanofluid fuels at each Al concentration were conducted over ten runs. Figure 5 shows that the density linearly varied with the Al concentration. The relationship between them is as follows:

(2) $$\rho_{nf}=\rho_{bf}(1+0.0056\varphi )(R^{2}=0.98)$$

where *ρ_nf_* and *ρ_bf_* are the densities of the nanofluid fuel and base fuel, respectively, and *φ* is the Al concentration.

**Figure 6:**
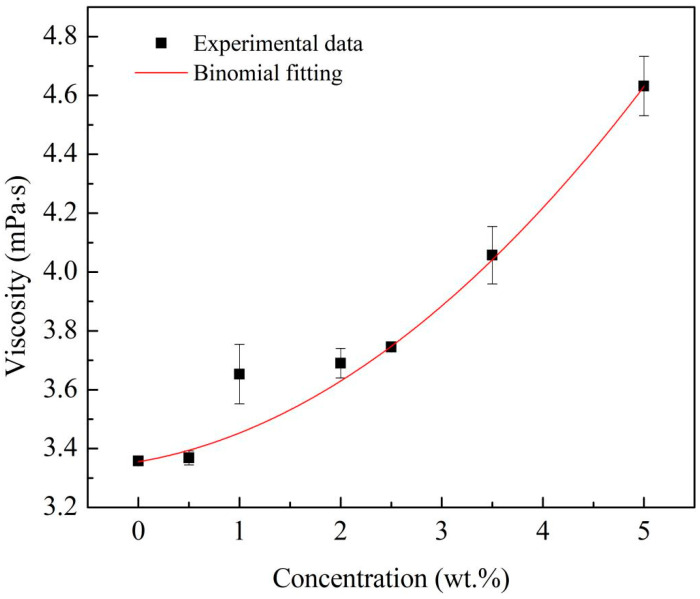
Viscosity of Al/JP-10/oleic acid nanofluid fuels at different Al concentrations.

At different concentrations, the viscosity of the Al/JP-10/oleic acid nanofluid fuels was repeatedly measured and the results are shown in Figure 6. When the mass fraction of the Al nanoparticles was 0.5 wt.%, the viscosity of the nanofluid fuel approximated that of the base fuel. At Al concentrations of 0.5~5.0 wt.%, the viscosity of the nanofluid fuels increased significantly. The viscosity is a quadratic function of Al concentration, which can be expressed as:

(3) $$\mu_{nf}=\mu_{bf}(1+0.0175\varphi +0.0117\varphi^{2})(R^{2}=0.99)$$

where *μ_nf_* and *μ_bf_* are the viscosity of the nanofluid fuel and base fuel, respectively.

The viscosity responds to the volume stress system of the nanofluid fuels and the coupled or interactive stable thermodynamic forces between nanoparticles. Chen et al. [24] explained that the thermodynamic force not only directly affects the volume stress, but indirectly affects the volume stress by changing the relative position of the nanoparticles. The direct and indirect contributions to the volume stress relate to the first-order (*φ*) and second-order (*φ*^2^) terms in Equation (3), respectively.

**Figure 7:**
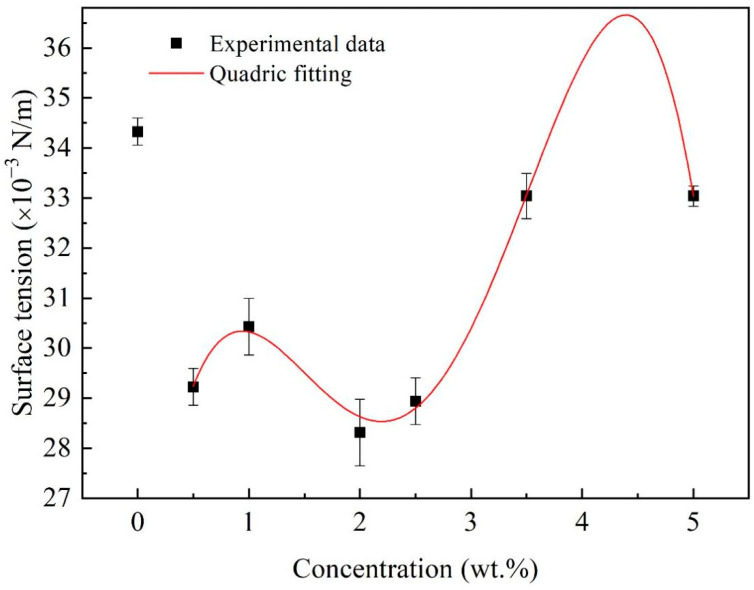
Surface tension of Al/JP-10/oleic acid nanofluid fuels at different Al concentrations.

Multiple measurements of the surface tension of the nanofluid fuels are shown in Figure 7. It is noticed that the surface tension of the nanofluid fuels with Al concentrations of 0.5~5.0 wt.% is lower than that of the base fuel, suggesting that the Al nanoparticles tend to accumulate on the liquid/gas interface and decrease the surface tension [25,26]. For the nanofluid fuels, with increasing Al concentrations, the surface tension oscillated, following the formula:

(4) $$\gamma_{nf}=23.6899+17.6578\varphi -15.4238\varphi^{2}+4.8894\varphi^{3}-0.4872\varphi^{4}(R^{2}=0.99)$$

### 3.3. Effect of Al Concentration on Evaporation Characteristics of Al/JP-10/Oleic Acid Nanofluid Fuels

#### 3.3.1. Evaporation Characteristics of JP-10

**Figure 8:**
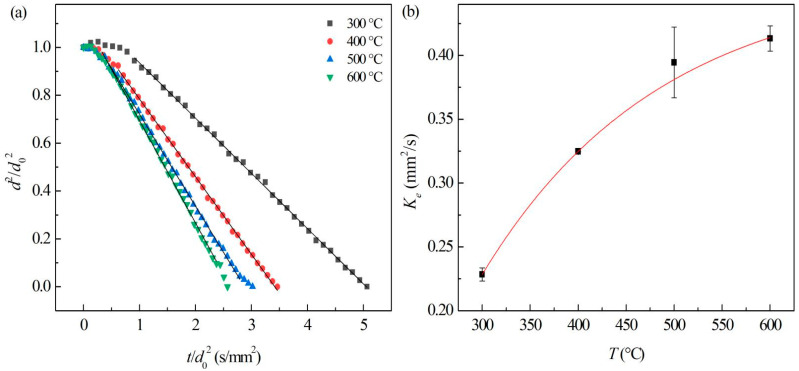
Evaporation of JP-10 at different ambient temperatures. (**a**) Variation of droplet diameter with the evaporation time, (**b**) evaporation rates at different ambient temperatures.

At different ambient temperatures in air, the evaporation process of JP-10 mainly underwent non-isothermal and isothermal evaporation stages (Figure 8a). During isothermal evaporation, the evaporation rates of JP-10 droplets follow the so-called *d*^2^-law [27], i.e.,:

(5) $$d^{2}=d_{0}^{2}-K_{e}t$$

In which *d*, *d*_0_ is the diameter and initial diameter of the droplets, and *t* and *K_e_* represent the evaporation time and evaporation rate constant, respectively.

By normalization, Equation (5) could be written as:

(6) $$d^{2}/d_{0}^{2}=1-K_{e}t/d_{0}^{2}$$

The evaporation rate constant can be obtained by the linearly fitting of $d^{2}/d_{0}^{2}\propto t/d_{0}^{2}$, shown as Figure 8b, which significantly increased with the temperature of ambient hot air (*T_air_*), and the relationship can be fitted as:

(7) $$K_{e}=0.4602-1.1628e^{-T_{air}/186.09}(R^{2}=1)$$

#### 3.3.2. Effect of Oleic Acid Concentration on Evaporation of JP-10

**Figure 9:**
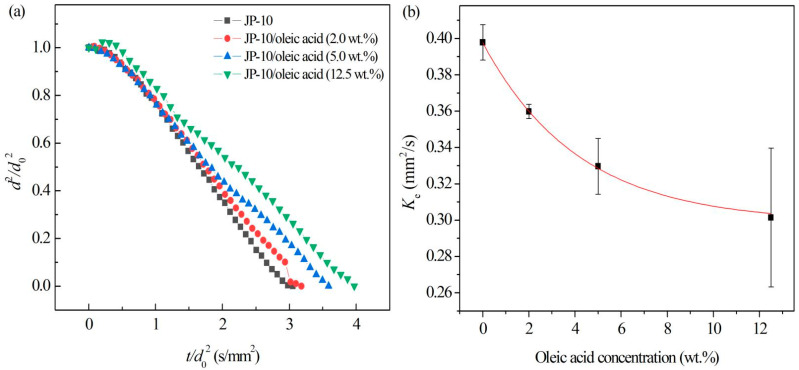
Evaporation of JP-10/oleic acid fuels at different oleic acid concentrations. (**a**) Variation of droplet diameter with the evaporation time, (**b**) evaporation rates at different oleic acid concentrations.

For JP-10 with the addition of oleic acid, the evaporation process (Figure 9) at 500 °C demonstrated the non-isothermal evaporation, isothermal evaporation, and microexplosion stages. It was observed (Figure 9a) that the isothermal evaporation of JP-10/oleic acid fuel was similar to that of JP-10, both of which followed the classical *d*^2^-law, but there was an additional fluctuating evaporation stage. Figure 9b shows that the evaporation rate decreased with increasing oleic acid concentration. The relationship between them can be fitted as:

(8) $$K_{e}=0.2954+0.1021e^{-\phi /4.478}(R^{2}=1)$$

where ϕ represents the oleic acid concentration. This reduction might be that the boiling point and volatility of JP-10 are lower than oleic acid. The experimental results were consistent with those reported by Javed et al. [27]. During evaporation, oleic acid coats the surface of the droplets and inhibits the evaporation of the JP-10, resulting in the reduction of the evaporation rate. Wang et al. [28] demonstrated that the evaporation life of the kerosene droplet became longer after adding cetyltrimethylammonium bromide (CTAB) surfactant, since the volatility of CTAB is lower than that of kerosene and its wrap on the surface hinders the evaporation of the kerosene droplet. Dai et al. [29] also discussed the phenomenon of the life of diesel droplets increasing slightly after adding the CTAB.

It worth noting that after adding the oleic acid into the JP-10, the droplets underwent microexplosions at the later stage of evaporation. Moreover, after increasing the content of oleic acid, the microexplosions became more intense. During evaporation, an oleic acid layer forms on the droplet surface. As the temperature inside the droplet gradually increases, the JP-10 of low boiling point volatilizes inside the droplet prior to the oleic acid, leading to local homogeneous nucleation, internal droplet gasification, and a pressure rise inside the droplet. If the surface tension of the droplet does not overcome enough of the pressure difference, the droplet breaks, leading to microexplosion [30].

#### 3.3.3. Effect of Al Concentration on Evaporation of Al/JP-10/Oleic Acid Nanofluid Fuel

**Figure 10:**
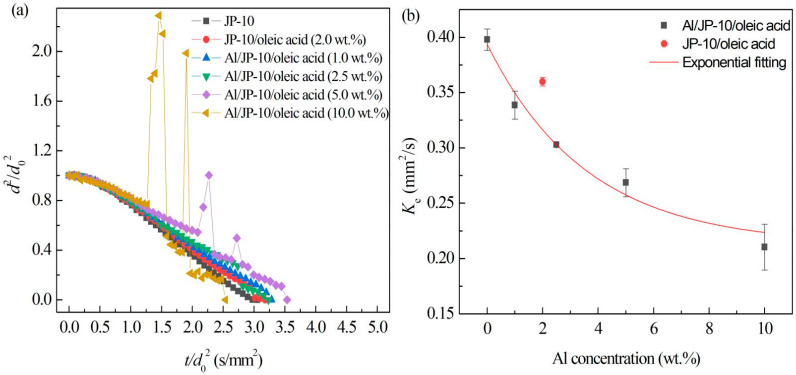
Evaporation of Al/JP-10/oleic acid nanofluid fuels at different Al concentrations. (**a**) Variation of droplet diameter with the evaporation time, (**b**) evaporation rates at different Al concentrations.

Figure 10a shows the evaporation data of Al/JP-10/oleic acid nanofluid fuel droplets with different Al concentrations (1.0 wt.%, 2.5 wt.%, 5.0 wt.% and 10.0 wt.%) at 500 °C. The content of oleic acid for each Al concentration is adopted to keep the best stability. That is, Al:oleic acid was 1:2 for below 5.0 wt.% Al concentration, 1:2.5 for 5.0 wt.% and 10.0 wt.%. It was clearly observed that, at 500 °C, the evaporation of Al/JP-10/oleic acid nanofluid fuel also included three stages, similar to JP-10/oleic acid fuel, namely, non-isothermal evaporation, isothermal evaporation, and microexplosion. With the increase in the Al concentration, the microexplosion phenomenon was more intense, and the microexplosion occurred earlier. The droplet underwent expansion and its diameter increased greatly before the microexplosion, and the diameter of the droplet decreased afterwards. There may be two mechanisms for the microexplosion after the addition of Al nanoparticles. One is that the ambient temperature is higher than the boiling point of JP-10, and the endothermic capacity of Al nanoparticles is greater than that of the base liquid, resulting in the temperature of the Al nanoparticles being greater than that of the base liquid, forming multiple heterogeneous nucleation sites. Inside the droplet, the base fuel around the Al nanoparticles is heated by the Al nanoparticles, forming gas-phase fuel. As a result, tiny bubbles are generated in the interior of the droplet, and such small bubbles aggregate into larger bubbles. Owing to the overheating of the bubble temperature, a microexplosion finally occurs [18,27,30]. The second is that the microexplosion is caused by the interaction of the added surfactant and Al nanoparticles. After adding surfactant, the droplet will undergo microexplosion, and with the increase in the surfactant concentration, the degree of microexplosion will be greater. In addition, the surfactant connects with the Al nanoparticles to form a more compact shell structure, which increases the intensity of the microexplosion [31]. Ao et al. [32] found that Al/kerosene droplets without surfactant did not undergo microexplosions.

Figure 10b demonstrates the average values and standard deviations of the fitting slopes of each group of droplets in the stable evaporation stage. The evaporation rate of JP-10 was the highest; the evaporation rate decreased after adding oleic acid, and further decreased after adding oleic acid and Al nanoparticles. With the increasing Al concentration, the evaporation rate of the droplets became lower. Their relationship is as follows:

(9) $$K_{e}=0.2123+0.1815e^{-\varphi /3.596}(R^{2}=0.98)$$

This indicates that the addition of oleic acid and Al nanoparticles inhibits the evaporation rate of the stable evaporation stage of the droplets. The reason for the decrease in the evaporation rate is that the addition of a high concentration of Al nanoparticles interacted with the surfactant, resulting in the accumulation of Al nanoparticles and the formation of a shell layer wrapped around the droplet surface [32], inhibiting the heat and mass transfer to the ambient hot air.

#### 3.3.4. Effect of Temperature on Evaporation of Al/JP-10/Oleic Acid Nanofluid Fuel

**Figure 11:**
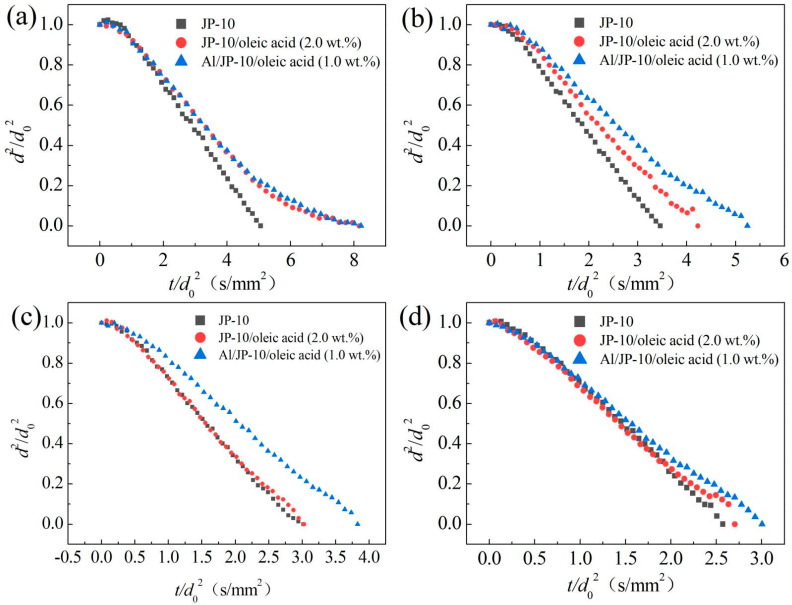
Evaporation of the JP-10, JP-10/oleic acid, Al/JP-10/oleic acid fuels at different ambient temperatures. (**a**) 300 °C, (**b**) 400 °C, (**c**) 500 °C, (**d**) 600 °C.

Figure 11 shows the evaporation data of the JP-10, JP-10/oleic acid (2.0 wt.%) and Al/JP-10/oleic acid (1.0 wt.% Al and 2.0 wt.% oleic acid) at 300–600 °C. The evaporation rate of JP-10 at each temperature was the highest. As the oleic acid and Al nanoparticles were added into the JP-10, the evaporation curves presented strong nonlinearity, especially at the later stage of evaporation. The droplets scarcely exhibited expansion or microexplosion at 300 °C, while microexplosion did occur at 400~600 °C. Since the temperature cannot meet the nucleation conditions, and there is no vaporized gas-phase fuel inside the droplet, microexplosion does not occur [32]. In addition, the evaporation of the droplets containing oleic acid and Al nanoparticles was rather slow in the later stage of evaporation (Figure 11a). This is because, with the evolution of evaporation, JP-10 is consumed gradually, and the concentration of oleic acid and Al nanoparticles becomes greater, significantly inhibiting the evaporation of the droplets. Therefore, the evaporation curves gradually flattened. At 400 °C, almost all of the fuel droplets maintained stable evaporation (Figure 11b). A slight microexplosion of droplets with oleic acid or Al nanoparticles was observed. The evaporation rates of the JP-10, JP-10/oleic acid, and Al/JP-10/oleic acid fuels evenly reduced in a sequence, indicating that oleic acid and Al nanoparticles largely contribute the reduction, respectively. At 500 °C, the evaporation rate of JP-10/oleic acid was close to that of JP-10, and the evaporation rate of the Al/JP-10/oleic acid nanofluid fuel was much lower than those of the JP-10 and JP-10/oleic acid fuels (Figure 11c). This suggests that the effect of oleic acid on the evaporation is compensated by the enhancement of ambient temperature. At such a temperature, the oleic acid scarcely affected the evaporation rate of JP-10; however, the addition of Al nanoparticles wrapped by oleic acid led to a reduction. At 600 °C, the evaporation rate of the Al/JP-10/oleic acid nanofluid fuel was approximated to those of the JP-10 and JP-10/oleic acid fuels (Figure 11d). This means that the enhancement of ambient temperature can increase the evaporation rates, compensating for the adverse effect of adding oleic acid and Al nanoparticles.

#### 3.3.5. Effect of Al Concentration on Microexplosion of Al/JP-10/Oleic Acid Nanofluid Fuel

**Figure 12:**
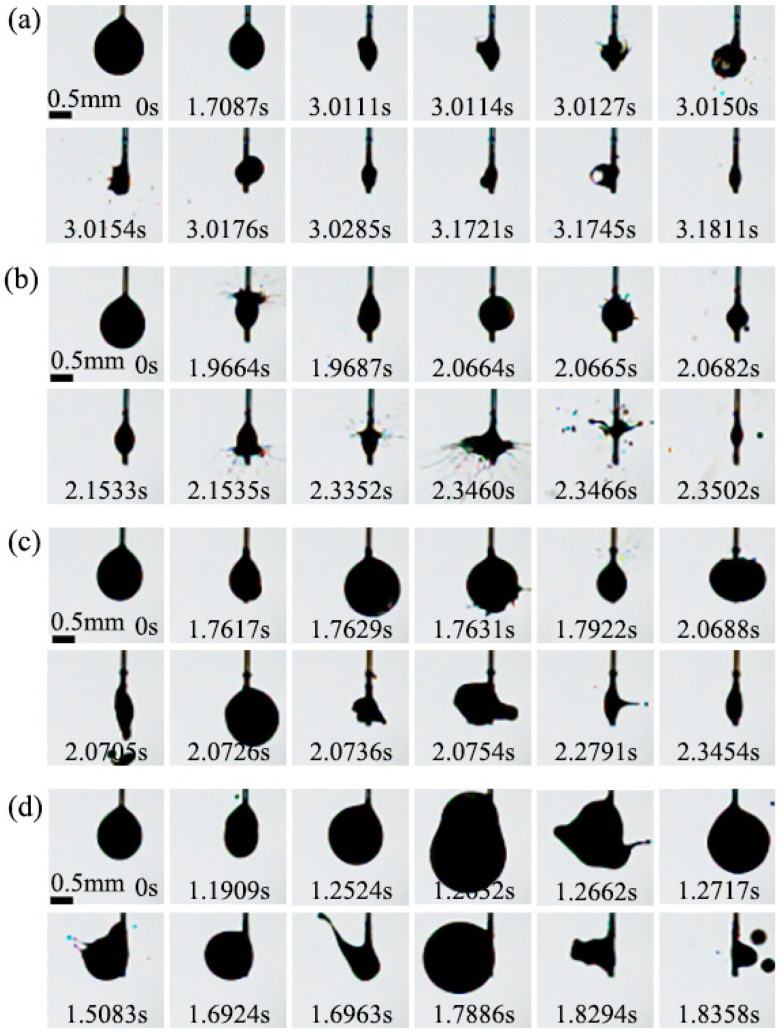
Microexplosion of Al/JP-10/oleic acid nanofluid fuel at different Al concentrations. (**a**) 1.0 wt.%, (**b**) 2.5 wt.%, (**c**) 5.0 wt.%, (**d**) 10.0 wt.%.

Since the droplets will undergo microexplosion after adding oleic acid and Al nanoparticles, it is necessary to evaluate the effect of Al concentration on the microexplosion behavior of the droplets. Microexplosion is an important part of improving the performance of nanofluid fuels. It can make the mother droplets disintegrate into smaller daughter droplets, i.e., secondary atomization, thus minimizing the droplet size and improving the evaporation rate and combustion efficiency. At 500 °C, the microexplosion of Al/JP-10/oleic acid nanofluid fuels with different Al concentrations (1.0 wt.%, 2.5 wt.%, 5.0 wt.%, 10.0 wt.%) are shown in Figure 12. The mass contents of oleic acid were set to keep the best dispersion stability of the Al nanoparticles into the base fuel. For the Al/JP-10/oleic acid nanofluid fuel of 1.0 wt.% (Figure 12a), the droplets evaporated till 3.0111 s, started to deform, and bubbles were generated inside the droplets. At 3.0114 s, the small droplets sprayed and then microexplosion followed. At 3.0150 s, the bubbles inside the droplets continuously expanded, splashing out microdroplets. At 3.0176 s and 3.1745 s, similar microexplosions occurred. The microexplosion of nanofluid fuel was more intense than that of JP-10/oleic acid, indicating that the addition of Al nanoparticles promotes the microexplosion. For the nanofluid fuel of 2.5 wt.% (Figure 12b), the microexplosion started at 1.9664 s, and occurred frequently as the evaporation proceeded. Before microexplosion, the bubbles formed and enlarged. As the pressure exceeded the surface tension of the droplet (Figure 7), the droplet experienced catastrophic fragmentation and produced quantities of tiny daughter droplets. For the nanofluid fuel of 5.0 wt.% (Figure 12c), the microexplosion firstly occurred at 1.7631 s, and the microexplosion process and phenomenon were similar. For the nanofluid fuel of 10.0 wt.% (Figure 12d), the moment of microexplosion was at 1.1909 s in advance, while there was a longer period of time between the droplet’s deformation to microexplosion, and the bubble size became larger compared to other nanofluid fuels of lower Al concentrations. This suggests that the Al nanoparticles formed a thicker shell at high concentrations to hinder the diffusion of gas-phase fuel and the breakup of droplets. It was found that, with the increase in the Al concentration, the timing of the microexplosions advanced at 3.0114 s, 1.9664 s, 1.7631 s and 1.1909 s for 1.0 wt.%, 2.5 wt.%, 5.0 wt.% and 10.0 wt.%, respectively. The bubble size before microexplosion became larger, and the degree of microexplosion was more intense.

## 4. Conclusions

In this paper, the stability, physical properties, evaporation, and microexplosion characteristics of Al/JP-10/oleic acid nanofluid fuel were measured, and the effects of adding different concentrations of oleic acid and Al nanoparticles on the evaporation and microexplosion characteristics at the temperature range of 300–600 °C were investigated. The conclusions can be made as follows:

(1) When the mass ratio of Al to oleic acid is 1:2, the dispersion stability of the nanofluid fuels is the best at Al concentrations of 1.0 wt.% and 2.5 wt.%, while when the mass ratio of Al to oleic acid becomes 1:2.5, the nanofluid fuel of 5.0 wt.% Al concentration can maintain the best stability.
(2) The density of the Al/JP-10/oleic acid nanofluid fuel increases linearly with the mass concentration of Al nanoparticles, and the viscosity coefficient is a quadratic function of the Al concentration. The surface tension of the nanofluid fuel oscillates as a quartic function of the Al concentration, lower than that of JP-10 at all concentrations.
(3) The evaporation of JP-10 includes non-isothermal and isothermal evaporation, while the JP-10/oleic acid and Al/JP-10/oleic acid fuels experience non-isothermal, isothermal evaporation, and microexplosion. The evaporation follows *d*^2^-law in the isothermal stage, and the evaporation rate decreases with the increasing concentrations of oleic acid and Al nanoparticles. The enhancement of the ambient temperature (300–600 °C) can significantly increase the evaporation rates of the fuels, compensating for the adverse effect of adding oleic acid and Al nanoparticles. At 600 °C, the evaporation rates of the JP-10, JP-10/oleic acid, Al/JP-10/oleic acid fuels are equivalent.
(4) At low ambient temperatures of 300 °C or below, the nanofluid fuels scarcely produce the microexplosion phenomenon. At high temperatures of 400 °C or above, the droplets undergo obvious microexplosion after adding oleic acid and Al nanoparticles. Moreover, with the increase in the Al concentration (1.0 wt.%, 2.5 wt.%, 5.0 wt.%, 10.0 wt.%), the time of microexplosion is advanced, the bubble size before microexplosion becomes larger, and the degree of microexplosion is more intense.

## Data Availability

The data presented in this study are available on request from the corresponding author.
